# A Pharmacogenetic Panel-Based Prediction of the Clinical Outcomes in Elderly Patients with Coronary Artery Disease

**DOI:** 10.3390/pharmaceutics16081079

**Published:** 2024-08-17

**Authors:** Lisha Dong, Shizhao Zhang, Chao Lv, Qiao Xue, Tong Yin

**Affiliations:** 1 Institute of Geriatrics, National Clinical Research Center for Geriatric Diseases, Second Medical Center of Chinese PLA General Hospital, Beijing 100853, China; lisadoctor2023@163.com (L.D.); zhangshizhao1997@163.com (S.Z.); lvch8822@163.com (C.L.); 2 Medical School of Chinese PLA, Chinese PLA General Hospital, Beijing 100853, China; 3 Department of Cardiology, First Medical Center of Chinese PLA General Hospital, Beijing 100853, China

**Keywords:** pharmacogenes, polypharmacy, panel, coronary artery diseases, elderly patients

## Abstract

Clinical annotations for the actionable pharmacogenetic variants affecting the efficacy of cardiovascular drugs have been collected, yet their impacts on elderly patients with coronary artery disease (CAD) undergoing polypharmacy remain uncertain. We consecutively enrolled 892 elderly patients (mean age 80.7 ± 5.2) with CAD and polypharmacy. All the included patients underwent genotyping for 13 variants in 10 pharmacogenes (*CYP2C19*, *CYP2C9*, *CYP4F2*, *CYP2D6*, *VKORC1*, *SLCO1B1*, *APOE*, *ACE*, *ADRB1*, and *MTHFR*), which have the clinical annotations for 12 drugs that are commonly prescribed for patients with CAD. We found that 80.3% of the elderly CAD patients had at least one drug–gene pair associated with a therapeutical drug change. After adjusting for covariates, the number of drug–gene pairs was independently associated with a decreased risk of both major cardiovascular events (MACEs) (adjusted hazard ratio [HR]: 0.803, 95% confidence interval [CI]: 0.683–0.945, *p* = 0.008) and all-cause mortality (adjusted HR: 0.848, 95% CI: 0.722–0.996, *p* = 0.045), but also with an increased risk of adverse drug reactions (ADRs) (adjusted HR: 1.170, 95% CI: 1.030–1.329, *p* = 0.016). The Kaplan–Meier survival curves showed that compared to patients without a drug–gene pair, a significantly lower risk of MACEs could be observed in patients with a drug–gene pair during a 4-year follow-up (HR: 0.556, 95% CI: 0.325–0.951, *p* = 0.013). In conclusion, the carrier status of the actionable drug–gene pair is predictive for the clinical outcomes in elderly patients with CAD and polypharmacy. Implementing early or preemptive pharmacogenetic panel-guided polypharmacy holds the potential to enhance clinical outcomes for these patients.

## 1. Introduction

Cardiovascular disease, specifically coronary artery disease (CAD), currently ranks first in terms of global mortality and morbidity [1]. Elderly people are more prone to CAD due to the key role of age in impairing the function of the cardiovascular system [2]. Although the age-adjusted CAD death rate is falling with the progress of healthcare technology, leading to a higher proportion of individuals living with CAD, the number of elderly CAD patients with multimorbidity and polypharmacy is increasing [3,4]. The concurrent use of at least five prescription medications is present in 30–50% of adults ≥65 years; 10–20% use ≥10 medications, and most elderly individuals will be on polypharmacy during their remaining lifespan [5]. Among these medications, cardiovascular drugs are the most widely used and the most frequent cause of adverse drug reactions in ambulatory elderly individuals [6]. Problematic polypharmacy is associated with an increase in adverse health outcomes including adverse drug reactions (ADRs), falls, functional impairment, frailty, increased length of hospital stays, readmissions, and mortality, which thus presents a significant challenge for personalized medication in elderly patients with CAD [3]. Variations in genes encoding for drug-metabolizing enzymes, drug transporters, and drug targets are widely recognized to contribute to pharmacokinetic (PK) and pharmacodynamic (PD) inter-patient differences in drug response [7]. Numerous studies have indicated strong evidence for the effect of actionable genotypes on the outcomes of vascular drugs [8]. In addition, more than 90% of the general population have at least one actionable genotype that is likely to influence their drug response, and over 60% of patients in primary care clinics are prescribed at least one drug with pharmacogenetic guidance [9]. It has been hypothesized that the use of a pharmacogenetic panel-based test will predict the response to multiple drugs over a patient’s lifetime, potentially improving drug-related outcomes, especially in elderly patients with polypharmacy [10]. To date, the majority of research on the efficacy of pharmacogenetic testing has focused on the association between a single drug–gene combination and the clinical outcomes [11]. The feasibility and clinical utility of panel-based genotyping to guide the prescription of polypharmacy in elderly patients remain uncertain [12]. The aim of this study was to explore the pharmacogenetic panel-based prediction of clinical outcomes in elderly CAD patients with polypharmacy.

## 2. Materials and Methods

### 2.1. Participants and Clinical Characteristics

This single-center observational cohort study consecutively included elderly patients aged ≥65 years old who were hospitalized for CAD and discharged with prescriptions of ≥5 medications in the Department of Cardiology, General Hospital of Chinese Peoples’s Liberation Army, from August 2018 to May 2022. Patients with severe renal or liver insufficiency (Child–Pugh class C liver disease or Kidney Disease Outcomes Quality Initiative Stage 5 chronic kidney disease), under organ replacement therapy, who failed to provide a blood sample, with severe diseases and an expected life expectancy <12 months, with invasive solid tumors or hematologic malignancies, or who experienced loss of follow-up within 1 year after study enrollment were excluded. Demographic information and medical data were extracted from the electronic health records. All included participants signed written informed consents before taking part in the study. The study was approved by the ethics committees of the People’s Liberation Army General Hospital (S2021-664-02).

### 2.2. Drug–Gene Pair Selection

A drug–gene pair was characterized by the clinical annotations associated with the pharmacogenetic variant, which had the actionable therapeutic recommendation supported by a minimum of Level 3 evidence from the Pharmacogenomics Knowledge Base (PharmGKB) [13]. Additionally, the minor allele frequency (MAF) of the variant was required to be at least 1% in East Asian populations, as reported by the 1000 Genomes Project [14]. Accordingly, drug–gene pairs were determined for each patient based on the panel of 13 genetic variants [CYP2C19*2 (rs4244285), CYP2C19*3 (rs4986893), CYP2C19*17 (rs12248560), CYP2C9*3 (rs1057910), CYP4F2*3 (rs2108622), CYP2D6*10 (rs1065852), *VKORC1* (rs9923231), SLCO1B1*5 (rs4149056), *APOE* (rs7412), *AOPE* (rs429358), *ACE* (rs1799752), *ADRB1* (rs1801253), and *MTHFR* (rs1801133)] of 10 genes associated with 12 drugs (clopidogrel, omeprazole, rabeprazole, warfarin, atorvastatin, rosuvastatin, fluvastatin, pravastatin, simvastatin, metoprolol, captopril, and folic acid) that are commonly prescribed for elderly patients with CAD. The clinical annotations for *APOE* genotypes were validated additionally according to the relevant literature [15]. The prevalence of the non-reference allele for *VKORC1* and *ADRB1* was up to 70% in the Chinese population. To reduce the impact of this high variability on drug–gene pairs in individuals, standard medication treatments was determined according to the individuals with the non-reference alleles. Drug–gene pairs were categorized into increased efficacy and decreased drug efficacy according to functional status. Increased efficacy indicates improved drug efficacy, toxicity, or lower required drug dosage. Conversely, decreased efficacy indicates reduced efficacy, toxicity, and a need for higher drug dosage. Details on the drug–gene pairs and related medications, gene, clinical annotations, and functional status are provided in Table S1.

### 2.3. Multiplexed Targeted Panel Genotyping

Nucleic acid mass spectrometry was employed to genotype the panel of 13 pharmacogenetic variants mentioned above. Genome DNA was isolated from the sediment of the peripheral blood cells for each individual and amplified through multiplex polymerase chain reaction (PCR). The amplicons underwent multiplex single-base extension (SBE) reactions following inactivation using shrimp alkaline phosphatase. The resulting SBE products were applied onto the SpectroCHIP II array and based on the MassARRAY Analyzer system using matrix-assisted laser desorption–ionization time-of-flight mass spectrometry (MALDI-TOF) technology. Genotypes at specified loci were identified using Typer software version 4.0 (Agena Biosciences, San Diego, CA, USA) in conjunction with SBE peak intensity analysis. Diplotypes for particular genes were inferred utilizing a haplotype translation table.

### 2.4. Clinical Outcomes

All enrolled patients received at least 1 year of follow-up. The clinical outcomes were collected by the investigators through electronic medical records and telephone communications. The primary clinical outcome was the incidence of major adverse cardiovascular events (MACEs), defined as a composite of cardiovascular mortality, nonfatal myocardial infarction, stent thrombosis, nonfatal stroke, and unplanned coronary revascularization. The secondary outcomes included all-cause mortality, adverse drug reactions (ADRs), and readmission. ADRs encompassed antithrombotic drug-related bleeding events as defined by the Bleeding Academic Research Consortium (BARC) criteria [16], statin-associated musculoskeletal symptoms according to the consensus of European Atherosclerosis Society Panel Statement on Assessment, Aetiology and Management [17], as well as drug-induced hypotension and falls in accordance with the 2019 American Geriatrics Society Beers Criteria^®^ [18].

### 2.5. Statistical Analysis

Continuous variables were presented as means ± standard deviations (SDs), and categorical variables were presented as frequencies (%) or medians with an interquartile range (IQR), respectively. The drug–gene pairs with effects on the clinical outcomes were summed up in a regression model. Association analyses between drug–gene pairs and clinical outcomes were performed using multivariate logistic regression with adjustment for age, gender, body mass index (BMI), number of comorbidities, coexisting diseases (hepatic and renal insufficiency, diabetes mellitus, heart failure, myocardial infarction), and number of drugs. Kaplan–Meier analysis was applied to construct the time-to-event curves for MACEs. The difference between the survival curves were compared with the log-rank test. The survival curves were analyzed using the log-rank test to assess differences. A two-sided *p* value of 0.05 was considered to indicate statistical significance. All statistical analyses were calculated using the R computing environment version 4.4.0.

## 3. Results

### 3.1. Baseline Clinical Characteristics

**Figure 1:**
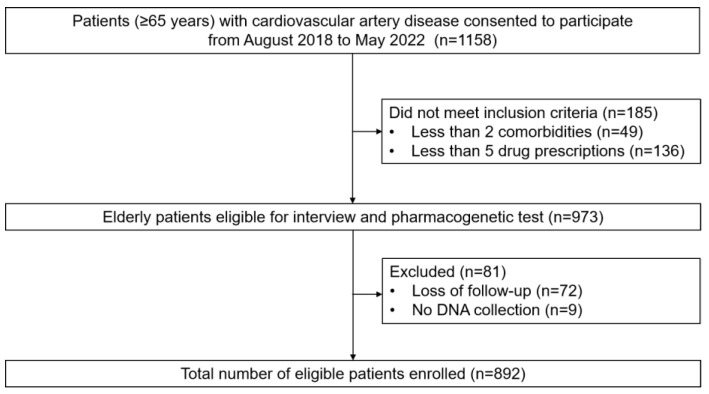
Flowchart of the study.

A total of 892 eligible patients were enrolled in the cohort (Figure 1). The mean age of the patients in the entire cohort was 80.7 ± 5.2 years (ranged from 65 to 97), with a male proportion of 61.8%. A total of 37.4% of the patients underwent percutaneous coronary intervention (PCI), and 17.9% had ≥5 comorbidities. The median number of oral prescriptions per patient at discharge was 7 (IQR 6–8, ranged from 5 to 13). The most common medications at discharge were lipid-lowering agents (93.8%), followed by antiplatelet agents (92.7%). The demographic characteristics of the study population are presented in Table 1.

### 3.2. Pharmacogenetic Variants and Genotypes

**Figure 2:**
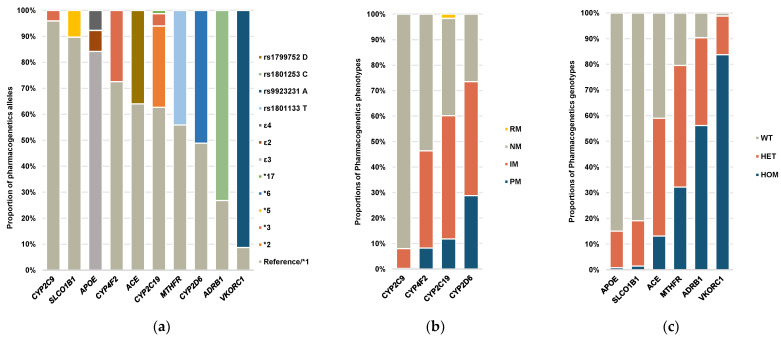
Pharmacogenetic allele, genotype, and phenotype proportions of the cohort for the 13 variants in 10 pharmacogenes. (**a**) Proportion of the different pharmacogenetic alleles (star (*) alleles) or variants. (**b**) Proportion of the genotype-based CYP metabolizer phenotypes. (**c**) Proportion of the non-CYP pharmacogene genotypes. WT, wild-type; HET, heterozygous; HOM, homozygous; NM, normal metabolizer; IM, intermediate metabolizer; PM, poor metabolizer; RM, rapid metabolizer.

The pharmacogenetic allele profiling of the elderly patients with CAD for the main 10 pharmacogenes is displayed in Figure 2a. The proportions of pharmacogenetic alleles were comparable with the data described for the East Asian in the 1000 Genome Project. The gene *VKORC1* exhibited the highest variability, with 91.3% of the alleles detected being the non-reference allele, followed by *ADRB1* (73.2%) and *CYP2D6* (51.1%). The allele, genotype, and phenotype frequency of the cohort for the 13 variants in 10 pharmacogenes are presented in Table S2. The genotype-based CYP metabolizer phenotypes for the pharmacogenes are shown in Figure 2b. More than 50% of the patients showed a different metabolizer status from the normal metabolism (NM) status for *CYP2C19* and *CYP2D6*, while more than 50% of the patients displayed an NM status for *CYP4F2* and *CYP2C9*. Among the non-CYP metabolic genes, more than 50% of the patients presented a heterozygous or homozygous status for *VKORC1*, *ADRB1*, *MTHFR*, and *ACE*, while more than 70% presented a wild-type status for *SLCO1B1* and *APOE* (Figure 2c).

### 3.3. Distribution of Drug–Gene Pairs

**Figure 3:**
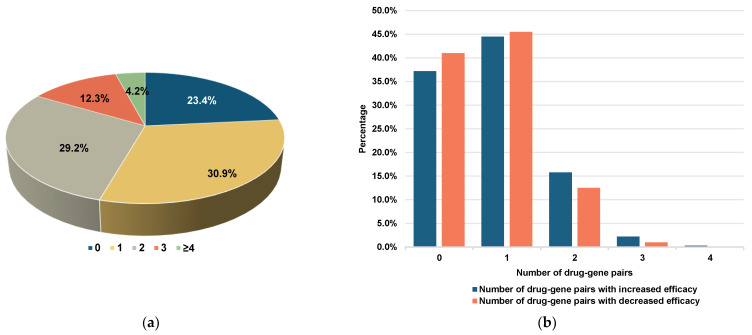
The distribution of drug–gene pairs. (**a**) The number of drug-gene pairs per patient. (**b**) The number of drug-gene pairs with increased efficacy and decreased efficacy per patient.

A total of 1404 drug–gene pairs were identified, with at least one drug–gene pair observed in 716 (80.3%) patients. Among these patients, 560 (62.8%) had at least one drug–gene pair with increased efficacy, while 526 (59.0%) had at least one drug–gene pair with decreased efficacy (Figure 3). The most common drug–gene pair with increased efficacy involved the *CYP2C19*-proton pump inhibitor (37.7%), followed by the *SLCO1B1*-statin (18.2%). Conversely, the most common drug–gene pair with decreased efficacy was *CYP2C19*-clopidogrel (38.0%), followed by *ADRB1*-metoprolol (20.5%) (Table S3). Figure S1 outlines the percentage of therapeutic recommendations according to the PharmGKB guideline in relation to drug–gene pairs. Notably, over half of the patients carried drug–gene pairs that were linked to the *CYP2C19*, *CYP2D6*, and *CYP4F2* genotypes.

### 3.4. Association between Drug–Gene Pairs and Clinical Outcomes

All included participants were followed for at least 1 year, with a median follow-up time of 39 months. During the follow-ups, the primary outcome of MACEs was recorded in 151 (16.9%) individuals. All-cause mortality occurred in 186 (20.8%) patients, ADRs in 260 (29.1%), and readmission in 410 (46.0%) patients. The incidence rates reported of ADRs were as follows: 15.9% for bleeding events, 14.0% for falls, 4.4% for statin-associated musculoskeletal symptoms, and 1.2% for hypotension.

The univariate analysis conducted in this study revealed a significant association between the number of drug–gene pairs and MACEs (hazard ratio [HR]: 0.821, 95% confidence interval [CI]: 0.700–0.962, *p* = 0.015) as well as ADRs (HR: 1.150, 95% CI: 1.016–1.302, *p* = 0.027). Additionally, a borderline association was observed with all-cause mortality. When we categorized the drug–gene pairs according to functional status, a significant association was found between the number of drug–gene pairs with increased efficacy and the outcomes of both MACEs (HR: 0.788, 95% CI: 0.623–0.996, *p* = 0.046) and ADRs (HR: 1.219, 95% CI: 1.017–1.460, *p* = 0.032). A borderline association was found between the number of drug–gene pairs with decreased efficacy and the outcomes of both all-cause mortality and MACEs (Table 2). 

After adjusting for covariates, the number of drug–gene pairs was independently associated with MACEs (adjusted HR: 0.803, 95% CI: 0.683–0.945, *p* = 0.008). In analysis of drug-gene pairs based on their functional status, both the number of drug–gene pairs with increased efficacy (adjusted HR: 0.777, 95% CI: 0.611–0.989, *p* = 0.044) and the number of drug–gene pairs with decreased efficacy (adjusted HR: 0.761, 95% CI: 0.586–0.989, *p* = 0.041) were significantly associated with a lower incidence rate of MACEs. The number of drug–gene pairs was also independently associated with a lower risk of all-cause mortality (adjusted HR: 0.848, 95% CI: 0.722–0.996, *p* = 0.045) and a higher risk of ADR (adjusted HR: 1.170, 95% CI: 1.030–1.329, *p* = 0.016). According to the function of drug–gene pairs, the number of drug–gene pairs with decreased efficacy was significantly associated with a decreased rate of all-cause mortality (adjusted HR: 0.704, 95% CI: 0.540–0.918, *p* = 0.010), and the number of drug–gene pairs with increased efficacy was associated with a higher incidence rate of ADRs (adjusted HR: 1.235, 95% CI: 1.026–1.485, *p* = 0.025). As for readmission, a significant contribution was found only for the number of drug–gene pairs with decreased efficacy (adjusted HR: 1.222, 95% CI: 1.010–1.478, *p* = 0.039) (Table 2). 

The association between drug–gene pairs formed by each specific drug and the clinical outcomes exhibited a consistent trend with the expected results of the whole study (Table S4). Sensitivity analyses were performed by employing different models of clinical covariance adjustment. The findings exhibited consistency across various analytical models for the association between drug–gene pairs and MACEs, as well as ADRs (Table S5). 

**Figure 4:**
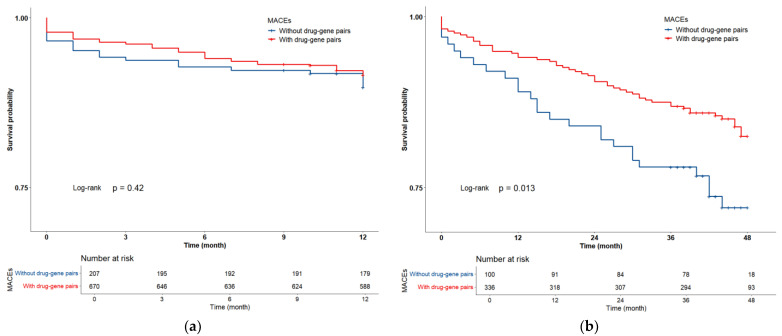
Kaplan–Meier curve of MACEs during 1-year follow-up (**a**) and 4-year follow-up (**b**). Major adverse cardiovascular events (MACEs) include cardiovascular mortality, nonfatal myocardial infarction, stent thrombosis, nonfatal stroke, and unplanned revascularization.

There was no significant difference in the clinical baseline between the groups with and without drug–gene pairs (Table S6). The Kaplan–Meier analysis showed that a significantly lower MACEs rate was found in drug–gene pair carriers compared to non-carriers during the 4-year follow-up (HR: 0.556, 95% CI: 0.325–0.951, *p* = 0.013). However, no significant difference in MACEs rates was observed within a 1-year follow-up (Figure 4).

## 4. Discussion

The main findings of the present study indicated that the high prevalence of drug–gene pairs among elderly patients with CAD might serve as a predictive indicator for the clinical outcomes, particularly in patients undergoing prolonged treatment with medications. As we know, this study represented the first attempt to assess the impacts of drug–gene pairs on the clinical outcomes in elderly patients with CAD and polypharmacy. It suggests that pharmacogenetic panel-based genotyping could facilitate personalized medication, especially in elderly patients with long-term polypharmacy.

Elderly patients with polypharmacy have been identified as an appealing group for pharmacogenetic panel-based genotyping to optimize medication precision [19]. Pharmacogenetic medications are commonly prescribed for elderly patients with polypharmacy, and it has been found that an actionable genetic variant can often be observed in elderly individuals. Consistent with previous publications, our study showed that 100% of the included elderly patients with CAD had at least one actionable pharmacogenetic variant, and 80.3% exhibited at least one relevant drug–gene pair [20,21]. As expected, the drugs related with the more variable genes showed the highest proportion of therapeutical changes [22]. The considerable variability in drug responses might necessitate therapeutic modifications for a significant proportion of elderly individuals [12].

Our investigation uncovered a negative relationship between the number of drug–gene pairs and MACEs, as well as mortality, while a positive correlation was observed between the number of drug–gene pairs and ADRs in elderly patients with CAD and polypharmacy. There is a scarcity of research exploring the association between drug–gene pairs and the clinical outcomes of MACEs and death in elderly patients with CAD and polypharmacy. The association identified between drug–gene pairs and ADRs is in line with the results of previous investigations [23,24,25]. Therefore, we speculated that the elderly patients with more drug–gene pairs might be more prone to experiencing ADRs due to the influence of the actionable genotypes on the drugs, potentially leading to an increased frequency of medication adjustments or dosage titration [26].

The routine adjustment of pharmacotherapy informed by pharmacogenetic data may reduce the occurrence of ADRs, particularly in elderly patients who are more susceptible to receiving inappropriate prescriptions. A recent meta-analysis observed that medication modification was more prevalent among elderly patients who underwent pharmacogenetic testing in comparison to those who received standard treatment [26]. It indicated that healthcare professionals could utilize pharmacogenomic knowledge to assist elderly patients in improving their therapeutic outcomes by eliminating potentially ineffective medication and replacing medications with unfavorable profiles. Despite the potential increase in ADRs in patients with more drug–gene pairs, the personalized medication approach resulting from this could potentially lower the risk of MACEs and overall mortality in elderly CAD patients being treated with multiple medications. Our subgroup analysis confirmed that an increased number of drug–gene pairs that are associated with increased efficacy was significantly linked to a higher likelihood of ADRs and a decreased incidence of MACEs. A higher age is associated with increased blood concentrations of drugs, altered metabolism, and increased risk of ADRs [27]. Consequently, elderly patients with drug–gene pairs exhibiting increased efficacy were more likely to have an elevated occurrence of ADRs [28]. Adjusting their prescribed medications in response to these ADRs could potentially reduce the risk of MACEs. The observation indicated that healthcare providers might consider reducing drug dosages for elderly patients with drug–gene pairs that increase drug efficacy or toxicity. It is important to note that while there may be an increase in ADRs and a decrease in MACEs for patients with drug–gene pairs exhibiting increased efficacy, this benefit may be offset by the risk of all-cause mortality [29,30]. In addition, our investigation revealed a negative association between the number of decreased-efficacy-related drug–gene pairs and MACEs, as well as mortality. The presence of the drug–gene pairs with decreased efficacy was similar to the drug dosage reduction in the elderly patients. Therefore, the therapeutic dosages of cardiovascular drugs for elderly patients with CAD should be down-regulated accordingly.

Although the passive or reactive regulation of drug type or dosage does occur even when the carrier status of the actionable drug–gene pairs is unknown, the clinical benefits of drug titration for reducing MACEs in carriers of drug–gene pairs could only be observed during long-term (4-year) drug therapy but not within 1 year in the elderly patients. Recently, the high-quality evidence of the PREPARE study showed that early or pre-emptive panel-based genotyping-guided treatments could result in a 30% reduction in ADRs [31]. Additionally, a post hoc analysis in the TAILOR-PCI trial demonstrated a significant reduction in the primary endpoint, mainly within the first 3 months under genotype-guided antiplatelet treatment in patients with PCI [32]. Therefore, we speculated that drug adjustments according to early or preemptive pharmacogenetic testing could be beneficial for the prevention of ADRs and MACEs at an earlier stage of drug therapy in elderly patients with CAD [33].

This study has several limitations that merit discussion. Firstly, it was a single-center observational study, and the results might be influenced by chance due to the relatively small sample size. Secondly, there is a potential bias that might prevent the fair generalization of the study’s findings to other patients with CAD, under the age of 65 and in non-Asian populations. Thirdly, the study focused on a pharmacogenetic panel test of commonly prescribed cardiovascular medications and its impact on clinical outcomes. The potential impact of such a test could be more significant if all drugs were considered; however, defining efficacy and safety endpoints for all drugs poses challenges. Fourthly, the study did not explore the potential benefits of the pharmacogenetic panel test for individual drugs, as the primary objective was to prospectively evaluate a comprehensive pharmacogenetics test panel encompassing numerous drugs. Fifthly, the variants in the *ABCB1* and *CES1* genes, especially *CES1* rs2244613, may influence the plasma concentrations of direct oral anticoagulants (DOACs) and the occurrence of bleeding side effects [34], with the level of evidence 3 on PharmGKB. In the present study, the observed clinical outcomes focused on drug efficacy and side effects. However, the available evidence linking *CES1* rs2244613 to bleeding events associated with DOACs remains limited and controversial, especially within the Asian population [35]. Therefore, although 10% of the study population received treatment with DOACs, we have chosen not to incorporate these genetic variants into the present panel. Given the relatively extensive use and potential bleeding side effects of DOACs in elderly patients with CAD, it might be beneficial to investigate the impact of pharmacogenes on the clinical outcomes of DOACs in future studies. Lastly, while drug–drug interactions are known to affect drug pharmacokinetics, they were not integrated into the model. Subsequent research should include drug–drug interactions and drug–gene pair associations as the quality of reference data advances.

## 5. Conclusions

The carrier status of drug–gene pairs serve as a predictive factor for the clinical outcomes in elderly patients with CAD. Pharmacogenetic panel-based genotyping, particularly when conducted early or preemptively, has the potential to improve clinical outcomes and thereby drive towards personalized or precision medicine in elderly patients with CAD and polypharmacy.

## Figures and Tables

**Table 1:** Baseline characteristics of participants in the cohort study.

Characteristics	n = 892
**Demographics**	
Gender (Male), n (%)	551 (61.8%)
Age (years), mean (SD)	80.7 (5.2)
BMI (kg/m^2^), Mean (SD)	24.4 (3.6)
Smoking, n (%)	88 (9.9%)
Drinking, n (%)	166 (18.6%)
**Comorbidities**	
Number of comorbidities, n (%)	
2	239 (26.8%)
3	283 (31.8%)
4	210 (23.5%)
≥5	160 (17.9%)
Myocardial infarction, n (%)	131 (14.7%)
Heart failure, n (%)	73 (8.2%)
Hypertension, n (%)	720 (80.7%)
Diabetes mellitus, n (%)	378 (42.4%)
Hyperlipidemia, n (%)	210 (23.5%)
Stroke, n (%)	170 (19.1%)
Hepatic and renal insufficiency, n (%)	133 (14.9%)
PCI, n (%)	334 (37.4%)
**Drugs at discharge**	
Number of drugs/patient, median (IQR, range)	7 (6–8, 5–13)
Antiplatelet agents, n (%)	827 (92.7%)
Oral anticoagulation agents, n (%)	97 (10.9%)
Lipid-lowering agents, n (%)	837 (93.8%)
Calcium channel blockers, n (%)	403 (45.2%)
Beta blockers, n (%)	569 (63.8%)
ACEI, n (%)	39 (4.4%)
ARB, n (%)	280 (31.4%)
Diuretic, n (%)	263 (29.4%)
PPI, n (%)	562 (63.0%)
Folic acid, n (%)	47 (5.3%)

BMI, body mass index; PCI, percutaneous coronary intervention; ACEI, angiotensin-converting enzyme inhibitor; ARB, angiotensin II receptor blocker; PPI, proton pump inhibitor. n (%), number (percent) of patients; SD, standard deviation; IQR, interquartile range.

**Table 2:** Associations between the number of drug–gene pairs and clinical outcomes.

	All-Cause Mortality		MACEs		ADRs		Readmission	
	HR (95% CI)	*p* Value	HR (95% CI)	*p* Value	HR (95% CI)	*p* Value	HR (95% CI)	*p* Value
**Univariate analysis**								
Number of drug–gene pairs	0.869 (0.753–1.004)	0.057	0.821 (0.700–0.962)	0.015	1.150 (1.016–1.302)	0.027	1.076 (0.960–1.206)	0.208
Increased efficacy	0.894 (0.725–1.103)	0.297	0.788 (0.623–0.996)	0.046	1.219 (1.017–1.460)	0.032	1.001 (0.847–1.184)	0.987
Decreased efficacy	0.792 (0.626–1.002)	0.052	0.795 (0.616–1.027)	0.079	1.133 (0.927–1.386)	0.223	1.211 (1.006–1.458)	0.043
**Multivariable analysis**								
Number of drug–gene pairs	0.848 (0.722–0.996)	0.045	0.803 (0.683–0.945)	0.008	1.170 (1.030–1.329)	0.016	1.074 (0.955–1.208)	0.232
Increased efficacy	0.933 (0.739–1.179)	0.563	0.777 (0.611–0.989)	0.044	1.235 (1.026–1.485)	0.025	0.990 (0.833–1.176)	0.905
Decreased efficacy	0.704 (0.540–0.918)	0.010	0.761 (0.586–0.989)	0.041	1.161 (0.945–1.425)	0.155	1.222 (1.010–1.478)	0.039

Multivariable logistic regression adjusted by age, gender, BMI, number of comorbidities, coexisting diseases (myocardial infarction, heart failure, diabetes mellitus, hepatic and renal insufficiency), and number of comedications. Major adverse cardiovascular events (MACEs) include cardiovascular mortality, nonfatal myocardial infarction, stent thrombosis, nonfatal stroke, and unplanned revascularization. Adverse drug reactions (ADRs) include bleeding, fall, hypotension, and statin-associated musculoskeletal symptoms. HR, hazard ratio; CI, confidence interval.

## Data Availability

The data presented in this study are available on request from the corresponding author due to privacy.

## Supplementary Materials

The following supporting information can be downloaded at https://www.mdpi.com/article/10.3390/pharmaceutics16081079/s1: Figure S1: Therapeutic recommendations for drug–gene pairs. Table S1: Selected drug–gene pairs related to clinical annotations and functional status. Table S2: Allele, genotype, and phenotype frequency of the cohort for the 13 variants in 10 pharmacogenes. Table S3: Drug–gene pair frequency of the cohort. Table S4: The impact of specific drug–gene pairs on the clinical outcomes. Table S5: Sensitivity analyses for the impact of drug–gene pairs on the clinical outcomes. Table S6: Baseline characteristics of the patients with and without drug–gene pairs.

## References

[1] Tsao C.W., Aday A.W., Almarzooq Z.I., Anderson C.A.M., Arora P., Avery C.L., Baker-Smith C.M., Beaton A.Z., Boehme A.K., Buxton A.E. (2023). Heart Disease and Stroke Statistics—2023 Update: A Report from the American Heart Association. Circulation.

[2] Ciumărnean L., Milaciu M.V., Negrean V., Orăs O.H., Sălăgean O., Ilu S., Vlaicu S.I. (2021). Cardiovascular Risk Factors and Physical Activity for the Prevention of Cardiovascular Diseases in the Elderly. Int. J. Environ. Res. Public Health.

[3] Mehta R.S., Kochar B.D., Kennelty K., Ernst M.E., Chan A.T. (2021). Emerging approaches to polypharmacy among older adults. Nat. Aging.

[4] Shah N.S., Xi K., Kapphahn K.I., Srinivasan M., Au T., Sathye V., Vishal V., Zhang H., Palaniappan L.P. (2022). Cardiovascular and Cerebrovascular Disease Mortality in Asian American Subgroups. Circ. Circ. Cardiovasc. Qual. Outcomes.

[5] Tamargo J., Kjeldsen K.P., Delpón E., Semb A.G., Cerbai E., Dobrev D., Savarese G., Sulzgruber P., Rosano G., Borghi C. (2022). Facing the challenge of polypharmacy when prescribing for older people with cardiovascular disease. A review by the European Society of Cardiology Working Group on Cardiovascular Pharmacotherapy. Eur. Heart J. Cardiovasc. Pharmacother..

[6] Schwartz J.B., Schmader K.E., Hanlon J.T., Abernethy D.R., Gray S., Dunbar-Jacob J., Holmes H.M., Murray M.D., Roberts R., Joyner M. (2018). Pharmacotherapy in Older Adults with Cardiovascular Disease: Report from an American College of Cardiology, American Geriatrics Society, and National Institute on Aging Workshop. J. Am. Geriatr. Soc..

[7] Pirmohamed M. (2023). Pharmacogenomics: Current status and future perspectives. Nat. Rev. Genet..

[8] Ross S., Krebs K., Paré G., Milani L. (2023). Pharmacogenomics in Stroke and Cardiovascular Disease: State of the Art. Stroke.

[9] Haidar C.E., Crews K.R., Hoffman J.M., Relling M.V., Caudle K.E. (2022). Advancing Pharmacogenomics from Single-Gene to Preemptive Testing. Annu. Rev. Genom. Hum. Genet..

[10] Cavallari L.H., Johnson J.A. (2023). Use of a multi-gene pharmacogenetic panel reduces adverse drug effects. Cell Rep. Med..

[11] Luzum J.A., Petry N., Taylor A.K., Van Driest S.L., Dunnenberger H.M., Cavallari L.H. (2021). Moving Pharmacogenetics Into Practice: It’s All About the Evidence!. Clin. Pharmacol. Ther..

[12] O’Shea J., Ledwidge M., Gallagher J., Keenan C., Ryan C. (2022). Pharmacogenetic interventions to improve outcomes in patients with multimorbidity or prescribed polypharmacy: A systematic review. Pharmacogenom. J..

[13] Pharmacogenetics and Pharmacogenomics Knowledge Base (PharmGKB) Drug Label Annotations. https://www.pharmgkb.org/labelAnnotations.

[14] The 1000 Genomes Project. https://www.internationalgenome.org/.

[15] Bennet A.M., Di Angelantonio E., Ye Z., Wensley F., Dahlin A., Ahlbom A., Keavney B., Collins R., Wiman B., de Faire U. (2007). Association of apolipoprotein E genotypes with lipid levels and coronary risk. JAMA.

[16] Mehran R., Rao S.V., Bhatt D.L., Gibson C.M., Caixeta A., Eikelboom J., Kaul S., Wiviott S.D., Menon V., Nikolsky E. (2011). Standardized bleeding definitions for cardiovascular clinical trials: A consensus report from the Bleeding Academic Research Consortium. Circulation.

[17] Stroes E.S., Thompson P.D., Corsini A., Vladutiu G.D., Raal F.J., Ray K.K., Roden M., Stein E., Tokgözoğlu L., Nordestgaard B.G. (2015). Statin-associated muscle symptoms: Impact on statin therapy-European Atherosclerosis Society Consensus Panel Statement on Assessment, Aetiology and Management. Eur. Heart J..

[18] 2019 American Geriatrics Society Beers Criteria^®^ Update Expert Panel (2019). American Geriatrics Society 2019 Updated AGS Beers Criteria® for Potentially Inappropriate Medication Use in Older Adults. J. Am. Geriatr. Soc..

[19] Uber R., Hayduk V.A., Pradhan A., Ward T., Flango A., Graham J., Wright E.A. (2023). Pre-emptive pharmacogenomics implementation among polypharmacy patients 65 years old and older. Pharmacogenomics.

[20] Jarvis J.P., Peter A.P., Keogh M., Baldasare V., Beanland G.M., Wilkerson Z.T., Kradel S., Shaman J.A. (2022). Real-World Impact of a Pharmacogenomics-Enriched Comprehensive Medication Management Program. J. Pers. Med..

[21] Kuch W., Rinner C., Gall W., Samwald M. (2016). How Many Patients Could Benefit From Pre-emptive Pharmacogenomic Testing and Decision Support? A Retrospective Study Based on Nationwide Austrian Claims Data. Stud. Health Technol. Inform..

[22] Nunez-Torres R., Pita G., Peña-Chilet M., López-López D., Zamora J., Roldán G., Herráez B., Álvarez N., Alonso M.R., Dopazo J. (2023). A Comprehensive Analysis of 21 Actionable Pharmacogenes in the Spanish Population: From Genetic Characterisation to Clinical Impact. Pharmaceutics.

[23] Eadon M.T., Rosenman M.B., Zhang P., Fulton C.R., Callaghan J.T., Holmes A.M., Levy K.D., Gupta S.K., Haas D.M., Vuppalanchi R. (2023). The INGENIOUS trial: Impact of pharmacogenetic testing on adverse events in a pragmatic clinical trial. Pharmacogenom. J..

[24] Stingl J.C., Kaumanns K.L., Claus K., Lehmann M.L., Kastenmuller K., Bleckwenn M., Hartmann G., Steffens M., Wirtz D., Leuchs A.K. (2016). Individualized versus standardized risk assessment in patients at high risk for adverse drug reactions (IDrug)-study protocol for a pragmatic randomized controlled trial. BMC Fam. Pract..

[25] Camargo A.C., Matte U., Botton M.R. (2023). Identification of adverse drug reactions that may be related to pharmacogenetics in a public hospital in the South of Brazil. Expert Opin. Drug Saf..

[26] David V., Fylan B., Bryant E., Smith H., Sagoo G.S., Rattray M. (2021). An Analysis of Pharmacogenomic-Guided Pathways and Their Effect on Medication Changes and Hospital Admissions: A Systematic Review and Meta-Analysis. Front. Genet..

[27] Hilmer S.N., McLachlan A.J., Le Couteur D.G. (2007). Clinical pharmacology in the geriatric patient. Fundam. Clin. Pharmacol..

[28] Le Couteur D.G., McLachlan A.J., de Cabo R. (2012). Aging, Drugs, and Drug Metabolism. J. Gerontol. Ser. A Biol. Sci. Med. Sci..

[29] Zazzara M.B., Palmer K., Vetrano D.L., Carfì A., Onder G. (2021). Adverse drug reactions in older adults: A narrative review of the literature. Eur. Geriatr. Med..

[30] Wester K., Jönsson A.K., Spigset O., Druid H., Hägg S. (2008). Incidence of fatal adverse drug reactions: A population based study. Br. J. Clin. Pharmacol..

[31] Swen J.J., van der Wouden C.H., Manson L.E., Abdullah-Koolmees H., Blagec K., Blagus T., Bohringer S., Cambon-Thomsen A., Cecchin E., Cheung K.C. (2023). A 12-gene pharmacogenetic panel to prevent adverse drug reactions: An open-label, multicentre, controlled, cluster-randomised crossover implementation study. Lancet.

[32] Pereira N.L., Farkouh M.E., So D., Lennon R., Geller N., Mathew V., Bell M., Bae J.H., Jeong M.H., Chavez I. (2020). Effect of Genotype-Guided Oral P2Y12 Inhibitor Selection vs. Conventional Clopidogrel Therapy on Ischemic Outcomes After Percutaneous Coronary Intervention: The TAILOR-PCI Randomized Clinical Trial. JAMA.

[33] Zhu Y., Moriarty J.P., Swanson K.M., Takahashi P.Y., Bielinski S.J., Weinshilboum R., Wang L., Borah B.J. (2021). A model-based cost-effectiveness analysis of pharmacogenomic panel testing in cardiovascular disease management: Preemptive, reactive, or none?. Genet. Med..

[34] Paré G., Eriksson N., Lehr T., Connolly S., Eikelboom J., Ezekowitz M.D., Axelsson T., Haertter S., Oldgren J., Reilly P. (2013). Genetic determinants of dabigatran plasma levels and their relation to bleeding. Circulation.

[35] Li H., Zhang Z., Weng H., Qiu Y., Zubiaur P., Zhang Y., Fan G., Yang P., Vuorinen A.L., Zuo X. (2022). Association between CES1 rs2244613 and the pharmacokinetics and safety of dabigatran: Meta-analysis and quantitative trait loci analysis. Front. Cardiovasc. Med..

