# Supplementary material for: A Pharmacogenetic Panel-Based Prediction of the Clinical Outcomes in Elderly Patients with Coronary Artery Disease
Source: Pharmaceutics. 2024 Aug 17;16(8):1079. doi: 10.3390/pharmaceutics16081079 (PMC11359157; doi:10.3390/pharmaceutics16081079)
Supplement: Supplementary file 1 [file pharmaceutics-16-01079-s001.zip › pharmaceutics-3136223-supplementary.pdf]

Supplementary Materials: A Pharmacogenetic Panel-Based Prediction of the Clinical Outcomes in Elderly Patients with Coronary Artery Disease

Lisha Dong, Shizhao Zhang, Chao Lv 1, Qiao Xue and Tong Yin

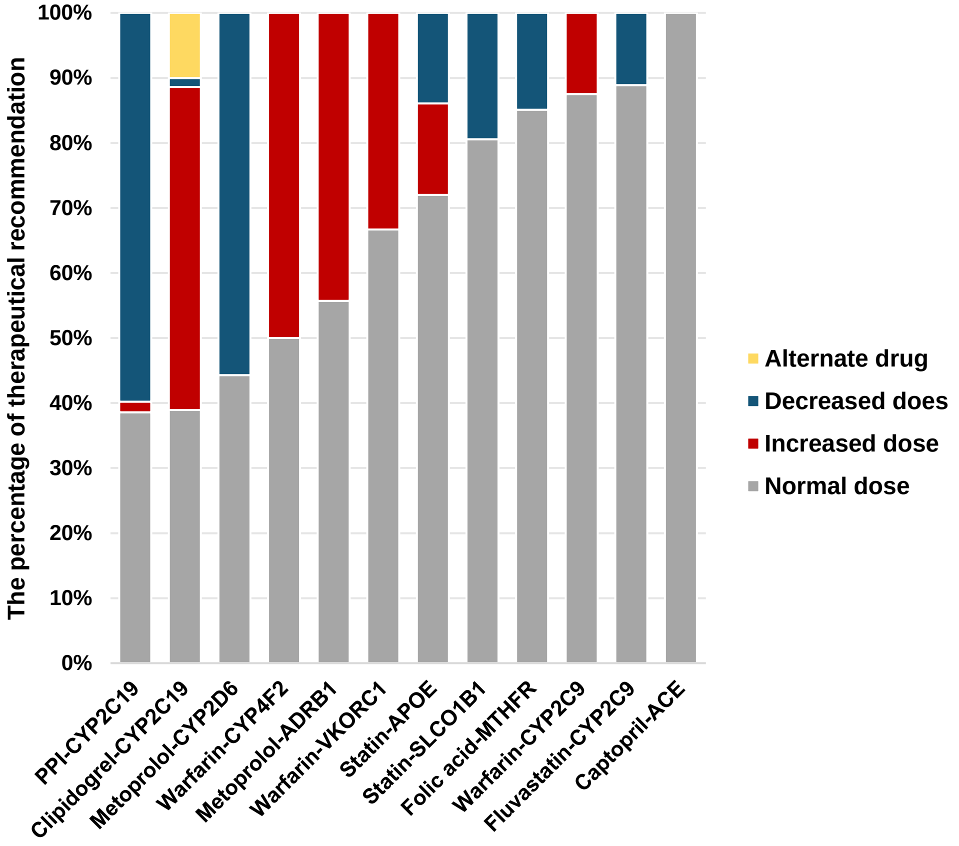

Figure S1. Therapeutical recommendations for drugs-gene pairs according to PharmGKB.

The therapeutical recommendations of the drug-gene pairs are depicted with a normal dose (gray), decreased dose (blue), increased dose (red), and alternate drug (yellow).

Table S1. Selected drug-gene pairs related to clinical annotations and functional status.

Drug	Gene	Nucleotide Variation	rs	Evidence levels (PharmGKB)	PharmGKB Clinical Annotation	Functional Status
Clopidogrel	*CYP2C19**2	c.681G>A	rs4244285	1	Patients carrying the *CYP2C19**2 allele in combination with a no, decreased, normal, or increased function allele who are treated with clopidogrel may have an increased risk for adverse cardiac and cerebrovascular events as compared to patients with two normal function alleles. However, conflicting evidence has been reported.	Decreased efficacy
	*CYP2C19**3	c.636G>A	rs4986893	1	Patients carrying the *CYP2C19**3 allele in combination with a no, decreased, normal, or increased function allele who are treated with clopidogrel may have an increased risk for adverse cardiac and cerebrovascular events as compared to patients with two normal function alleles.	Decreased efficacy
	*CYP2C19**17	c.-806C>T	rs12248560	1	Patients carrying the *CYP2C19**17 allele in combination with a no or decreased function allele who are treated with clopidogrel may have an increased risk for adverse cardiac and cerebrovascular events as compared to patients with two normal function alleles.	Increased efficacy
PPI	*CYP2C19**2	c.681G>A	rs4244285	1	Patients carrying the *CYP2C19**2 allele in combination with a no, decreased, normal, or increased function allele may have a better response to PPI (smaller % of time with intragastric pH < 4.0, a higher intragastric pH during a 24-hour time period, increased likelihood of H. pylori eradication, among other parameters) as compared to patients with two normal function alleles.	Increased efficacy
	*CYP2C19**3	c.636G>A	rs4986893	1	Patients carrying the *CYP2C19**3 allele in combination with a no, decreased, normal, or increased function allele may have a better response to PPI (smaller % of time with intragastric pH < 4.0, a higher intragastric pH during a 24-hour time period, increased likelihood of H. pylori eradication, among other parameters) as compared to patients with two normal function alleles.	Increased efficacy

	*CYP2C19**17	c.-806C>T	rs12248560	1	Patients carrying the *CYP2C19**17 allele in combination with a normal or increased function allele may have increased metabolism of omeprazole as compared to patients with two normal function alleles. Patients carrying the CYP2C19*17 allele in combination with a no or decreased function allele may have decreased metabolism of PPI as compared to patients with two normal function alleles.	Decreased efficacy
Warfarin	*CYP2C9**3	c.1075A>C	rs1057910	1	Patients carrying the *CYP2C9**3 allele in combination with a normal, decreased, or no function allele may require a lower dose of warfarin as compared to patients with two normal function alleles.	Decreased dosage
	*CYP4F2**3	c.1297G>A	rs2108622	1	Patients with the rs2108622 AG genotype may have increased warfarin dosage requirements as compared to patients with the GG genotype.<br>Patients with the rs2108622 AA genotype may have increased warfarin dosage requirements as compared to patients with the GG or AG genotype.	Increased dosage
	VKORC1	c.-1639G>A	rs9923231	1	Patients with the rs9923231 GG genotype may require an increased dose of warfarin as compared to patients with the AG or AA genotype.<br>Patients with the rs9923231 AG genotype may require a decreased dose of warfarin as compared to patients with the GG genotype or an increased dose as compared to patients with the TT genotype.	Increased dosage
Statin	*SLCO1B1**5	c.521T>C	rs4149056	1	Patients carrying SLCO1B1*5 allele in combination with a normal, no, or increased function allele may have a higher risk of statin-related myopathy when treated with simvastatin as compared to patients with two normal function alleles.	Increased toxicity
	APOE	c.526C>T<br>c.388T>C	rs7412<br>rs429358	/	Patients with the E2/E2 genotype may have increased response to statin as compared to patients with the E3/E3 or E2/E4 genotype.<br>Patients with the E2/E3genotype may have increased response to statin as compared to patients with the E3/E3 or E2/E4 genotype.	Increased efficacy
	APOE	c.526C>T<br>c.388T>C	rs7412<br>rs429358	/	Patients with the E3/E4 genotype may have decreased response to statin as compared to patients with the E3/E3 or E2/E4 genotype.<br>Patients with the E4/E4 genotype may have decreased response to statin as compared to patients with the E3/E3 or E2/E4 genotype.	Decreased efficacy
Fluvastatin	*CYP2C9**3	c.1075A>C	rs1057910	1	Patients carrying the *CYP2C9**3 allele in combination with a normal, decreased, or no function allele may have increased	Increased toxicity

..

					likelihood of adverse events when treated with fluvastatin as compared to patients with two normal function alleles.	
Metoprolol	*CYP2D6**10	c.100C>T	rs1065852	1	Patients carrying the *10 allele in combination with a decreased or no function allele may have decreased metabolism of metoprolol as compared to patients with alleles that result in a normal metabolizer phenotype.	Increased efficacy
	ADRB1	c.1165G>C	rs1801253	3	Patients with the GG genotype may have a decreased response to metoprolol as compared to patients with the CC genotype. Patients with the GC genotype may have a decreased response to metoprolol as compared to patients with the CC genotype.	Decreased efficacy
Folic acid	*MTHFR*	C677T	s1801133	3	Patients with the TT genotype may have decreased metabolism of folic acid as compared to patients with the CC genotype. Patients with the CT genotype may have decreased metabolism of folic acid as compared to patients with the CC genotype.	Increased efficacy
Captopril	*ACE*	I/D	rs1799752	2	Patients with the rs1799752 del/del genotype may have a decreased response when treated with captopril as compared to patients with the insert/del or insert/insert genotypes. Patients with the rs1799752 insert/del genotype and may have an increased response when treated with captopril as compared to patients with the del/del genotype, or a decreased response compared to patients with the insert/insert genotype	Decreased efficacy

Table S2. Allele, genotype and phenotype frequency of the cohort for the 13 variants in 10 pharmacogenes.

Gene	Alleles	Nucleotide Variation	rs	Allel Frequency	East Asian allele frequency	Diplotypes	Genotype/Phenotype	Genotype/Phenotype Frequency
CYP2C19	*2	c.681G>A	rs4244285	31.3%	31.3%	*2/*2, *3/*3, *2/*3	PM	11.8%
	*3	c.636G>A	rs4986893	4.7%	5.6%	*1/*2, *1/*3	IM	48.4%
	*17	c.-806C>T	rs12248560	1.3%	1.5%	*1/*17，*17/*17	RM	1.7%
CYP2C9	*3	c.1075A>C	rs1057910	4.2%	3.4%	*3/*3	PM	0.2%
						*1/*3	IM	7.8%
CYP4F2	*3	c.1297G>A	rs2108622	27.4%	21.4%	*3/*3	PM	8.2%
						*1/*3	IM	38.2%

VKORC1	A	c.-1639G>A	rs9923231	91.3%	88.5%	AA	HOM	83.8%
						GA	HET	15.0%
SLCO1B1	*5	c.521T>C	rs4149056	10.3%	12.3%	*5/*5	HOM	1.4%
						*1/*5	HET	17.7%
APOE	T	c.526C>T	rs7412	8.1%	10.0%	TT	HOM	1.0%
						CT	HET	14.1%
	C	c.388T>C	rs429358	7.7%	8.6%	CC	HOM	0.6%
						CT	HET	14.3%
CYP2D6	*10	c.100C>T	rs1065852	51.1%	57.1%	*10/*10	PM	28.8%
						*1/*10	IM	44.7%
ADRB1	C	c.1165G>C	rs1801253	73.2%	78.8%	CC	HOM	56.2%
						GC	HET	34.2%
MTHFR	T	C677T	rs1801133	55.9%	29.6%	TT	HOM	32.2%
						CT	HET	47.4%
ACE	/	I/D	rs1799752	/	/	DD	HOM	13.1%
						DI	HET	45.9%

Alleles frequency in the East Asian population reported in 1000 Genomes study (https://www.ncbi.nlm.nih.gov/snp).WT, wild-type; HET, heterozygous; HOM, homozygous; NM, normal metabolizer; IM, interme-diate metabolizer; PM, poor metabolizer; RM, rapid metabolizer.

Table S3. Drug-gene pairs frequency of the cohort.

Drug	Gene	Genotype/Phenotype	Number of drug-gene pairs	Drug-gene pairs frequency	Functional Status
Clopidogrel	*CYP2C19*	PM, IM	339	38.0%	Decreased efficacy
		RM	8	0.9%	Increased efficacy
PPI	*CYP2C19*	PM, IM	336	37.7%	Increased efficacy
		RM	9	1.0%	Decreased efficacy
Warfarin	*CYP2C9**3	IM	1	0.1%	Increased efficacy
	*CYP4F2**3	*3/*3, *1/*3	4	0.4%	Decreased efficacy
	VKORC1	GG, GA	3	0.3%	Decreased efficacy

Statin	*SLCO1B1**5	Poor function, Decreased function	162	18.2%	Increased efficacy
	APOE	E2/E2，E2/E3	51	5.7%	Increased efficacy
		E3/E4, E4/E4	57	6.4%	Decreased efficacy
Fluvastatin	*CYP2C9**3	IM	1	0.1%	Increased efficacy
Metoprolol	*CYP2D6**10	*10/*10	118	13.2%	Increased efficacy
	ADRB1	GG, GC	183	20.5%	Decreased efficacy
Folic acid	*MTHFR*	TT, CT	6	0.7%	Increased efficacy
Captopril	*ACE*	DD, DI	0	0.0%	Decreased efficacy

PM, poor metabolizer; IM, intermediate metabolizer; NM, normal metabolizer; RM, rapid metabolizer.

Table S4. The impact of specific drug-gene pair on the clinical outcomes. .

Drug-gene pair		MACEs		All-cause mortality		ADRs	
		HR (95% CI)	*P* Value	HR (95% CI)	*P* Value	HR (95% CI)	*P* Value
**Clopidogrel**	*CYP2C19*	0.570 (0.385-0.844)	0.005	0.784 (0.536-1.146)	0.209	1.051 (0.780-1.418)	0.742
PPI	*CYP2C19*	0.527 (0.352-0.788)	0.002	0.785 (0.531-1.160)	0.224	1.433 (1.060-1.938)	0.019
Statin	*SLCO1B1**5						
	APOE	0.841 (0.617-1.146)	0.273	0.764 (0.555-1.050)	0.097	1.308 (1.030-1.661)	0.028
Fluvastatin	*CYP2C9**3						
Metoprolol	*CYP2D6**10	1.022 (0.754-1.385)	0.889	0.929 (0.677-1.273)	0.645	1.058 (0.826-1.355)	0.657
	ADRB1						

"The relationship between clinical outcomes and the drug-gene pairs associated with folic acid, captopril and warfarin was not evaluated due to the insufficient number of elderly patients in the study.
Multivariable logistic regression adjusted by age, sex, BMI, number of comorbidities, coexisting diseases (myocardial infarction, heart failure, diabetes mellitus, hepatic and renal insufficiency) and number of comedications.

Major adverse cardiovascular events (MACEs) included cardiovascular mortality, nonfatal myocardial infarction, stent thrombosis, nonfatal stroke, and unplanned revascularization.

Adverse drug reactions (ADRs) included bleeding, fall, hypotension, and statin-associated musculoskeletal symptoms.

HR, hazard ratio; CI, confidence interval.PPI, proton pump inhibitor; PM, poor metabolizer; IM, intermediate metabolizer; NM, normal metabolizer; RM, rapid metabolizer."

Table S5. Sensitivity analyses for the impact of drug-gene pairs on clinical outcomes.

	**All-cause mortality**		**MACEs**		**ADRs**	
	HR (95% CI)	*P* value	HR (95% CI)	*P* value	HR (95% CI)	*P* value
**Sensitive analysis 1**						
Number of drug-gene pairs without PPI and folic acid	0.830 (0.683-1.009)	0.062	0.839 (0.692-1.019)	0.076	1.146 (0.983-1.335)	0.081
**Sensitive analysis 2**						
Basic model	0.869 (0.753-1.004)	0.057	0.821 (0.700-0.962)	0.015	1.150 (1.016-1.302)	0.027
Model 1	0.881 (0.757-1.026)	0.104	0.821 (0.700-0.964)	0.016	1.156 (1.019-1.312)	0.024
Model 2	0.848 (0.722-0.996)	0.045	0.803 (0.683-0.945)	0.008	1.170 (1.030-1.329)	0.016

Basic model did not adjust for any confounding factors.
Model 1 adjusted for gender, age, number of comorbidities and number of comedications.
Model 2 adjusted for age, gender, BMI, number of comorbidities, number of comedications and coexisting diseases (myocardial infarction, heart failure, diabetes mellitus, hepatic and renal insufficiency). Major adverse cardiovascular events (MACEs) included cardiovascular mortality, nonfatal myocardial infarction, stent thrombosis, nonfatal stroke, and unplanned revascularization.
Adverse drug reactions (ADRs) included bleeding, fall, hypotension, and statin-associated musculoskeletal symptoms.

Table S6. Baseline characteristics of the patients with and without drug-gene pairs.

Variables	With drug-gene pairs	Without drug-gene pairs	*P* Value
**Demographics**			
Gender (Male), n (%)	443 (49.7%)	108 (12.1%)	0.901
Age (years), mean (SD)	80.6 ± 5.1	80.9 ±5.5	0.147
BMI (kg/m2), Mean (SD)	24.4 ±3.6	24.4 ± 3.4	0.877
Smoking, n (%)	73 (8.2%)	15 (1.7%)	0.505
Drinking, n (%)	135 (15.1%)	31 (3.5%)	0.705
**Comorbidities**			
Number of comorbidities			0.055
2	203 (22.8%)	36 (4.0%)	
3	214 (24.0%)	69 (7.7%)	
4	165 (18.5%)	45 (5.0%)	
≥5	134 (15.0%)	26 (2.9%)	
Myocardial infarction, n (%)	108 (12.1%)	23 (2.6%)	0.499
Heart failure, n (%)	58 (6.5%)	15 (1.7%)	0.855
Hypertension, n (%)	574 (64.3%)	146 (16.4%)	0.401
Diabetes mellitus, n (%)	304 (34.1%)	74 (8.3%)	0.921
Hyperlipidemia, n (%)	171 (19.2%)	39 (4.4%)	0.629
Stroke, n (%)	135 (15.1%)	35 (3.9%)	0.755
Hepatic and renal insufficiency, n (%)	108 (12.1%)	25 (2.8%)	0.764
PCI, n (%)	272 (30.5%)	62 (7.0%)	0.498
**Drugs at discharge**			
Number of drugs/patient			0.081
5	138 (15.5%)	37 (4.1%)	
6	178 (20.0%)	53 (5.9%)	
7	147 (16.5%)	45 (5.0%)	
≥8	253 (28.4%)	41 (4.6%)	

BMI, body mass index; PCI, percutaneous coronary intervention; n (%), number (percent) of patients; SD, standard deviation; IQR, interquartile range.
